# 2-[(*E*)-(4-Bromo­phenyl)imino­methyl]-4-chloro­phenol

**DOI:** 10.1107/S1600536814000981

**Published:** 2014-02-05

**Authors:** Xiao-Li Gao, Si-Si Feng, Cai-Xia Yuan, Miao-Li Zhu

**Affiliations:** a Department of Chemistry, Taiyuan Normal College, Taiyuan, Shanxi 030031, People’s Republic of China; b Institute of Molecular Science, Key Laboratory of Chemical Biology and Molecular Engineering of the Education Ministry, Shanxi University, Taiyuan, Shanxi 030006, People’s Republic of China

## Abstract

In the title compound, C_13_H_9_BrClNO, the dihedral angle between the substituted benzene rings is 44.25 (11)°. There are strong intra­molecular O—H⋯N hydrogen bonds, which generate *S*(6) rings, and also inter­molecular Cl⋯Cl [3.431 (3) Å] and Br⋯ Br [3.846 (1) Å] contacts. The crystal packing a C—H⋯O and C—H⋯π inter­actions.

## Related literature   

For background to the biological activity of Schiff bases, see: Akmal *et al.* (2007 ▶); Li *et al.* (2007 ▶, 2011 ▶); Lu *et al.* (2011 ▶); Ma *et al.* (2011 ▶); Rehmana *et al.* (2008 ▶); Ritter *et al.* (2009 ▶); Vanco *et al.* (2008 ▶); Yuan *et al.* (2009 ▶, 2010 ▶). For related structures, see: Ardakani *et al.* (2011 ▶). For hydrogen-bond motifs, see: Bernstein *et al.* (1995 ▶).
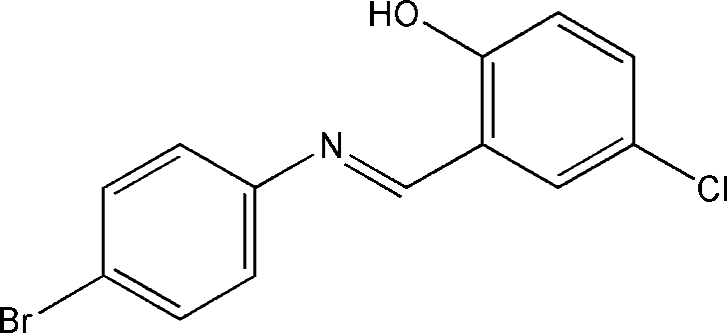


## Experimental   

### 

#### Crystal data  

C_13_H_9_BrClNO
*M* *_r_* = 310.57
Orthorhombic, 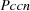

*a* = 6.9964 (15) Å
*b* = 55.786 (12) Å
*c* = 6.1443 (14) Å
*V* = 2398.1 (9) Å^3^
*Z* = 8
Mo *K*α radiation
μ = 3.63 mm^−1^
*T* = 298 K
0.30 × 0.25 × 0.20 mm

#### Data collection  

Bruker SMART 1K CCD area-detector diffractometer
Absorption correction: multi-scan (*SADABS*; Sheldrick, 2000 ▶) *T*_min_ = 0.409, *T*_max_ = 0.530
30095 measured reflections
3013 independent reflections
2056 reflections with *I* > 2σ(*I*)
*R*_int_ = 0.061

#### Refinement  

*R*[*F* ^2^ > 2σ(*F* ^2^)] = 0.054
*wR*(*F* ^2^) = 0.128
*S* = 1.14
3013 reflections
155 parameters
H-atom parameters constrained
Δρ_max_ = 0.42 e Å^−3^
Δρ_min_ = −0.95 e Å^−3^

### 

Data collection: *SMART* (Bruker, 2000 ▶); cell refinement: *SAINT* (Bruker, 2000 ▶); data reduction: *SAINT*; program(s) used to solve structure: *SHELXS97* (Sheldrick, 2008 ▶); program(s) used to refine structure: *SHELXL97* (Sheldrick, 2008 ▶); molecular graphics: *ORTEP-3 for Windows* (Farrugia, 2012 ▶); software used to prepare material for publication: *publCIF* (Westrip, 2010 ▶).

## Supplementary Material

Crystal structure: contains datablock(s) I, global. DOI: 10.1107/S1600536814000981/fj2653sup1.cif

Structure factors: contains datablock(s) I. DOI: 10.1107/S1600536814000981/fj2653Isup2.hkl

Supporting information file. DOI: 10.1107/S1600536814000981/fj2653Isup3.cml

CCDC reference:

Additional supporting information:  crystallographic information; 3D view; checkCIF report

## Figures and Tables

**Table 1:** Hydrogen-bond geometry (Å, °) *Cg*1 and *Cg*2 are the centroids of the C1–C6 and C8–C13 benzene rings, respectively.

*D*—H⋯*A*	*D*—H	H⋯*A*	*D*⋯*A*	*D*—H⋯*A*
O1—H1⋯N1	0.82	1.87	2.593 (4)	147
C2—H2⋯*Cg*1^i^	0.93	2.82	3.489 (5)	129
C5—H5⋯*Cg*1^ii^	0.93	2.85	3.513 (5)	129
C10—H10⋯*Cg*2^iii^	0.93	2.75	3.460 (5)	133
C13—H13⋯*Cg*2^iv^	0.93	2.78	3.473 (5)	132

Symmetry codes: (i) 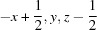; (ii) 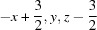; (iii) 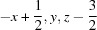; (iv) 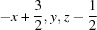.

**Fig. 1.**
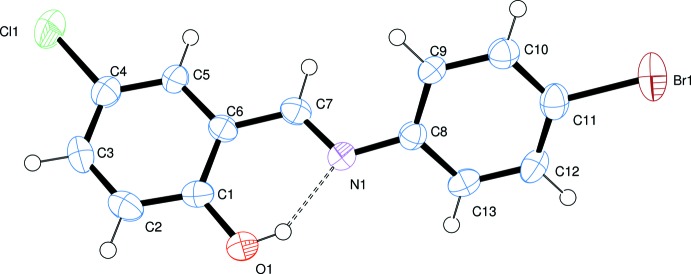
A view of the structure of (I), with displacement ellipsoids drawn at the 30% probability level. Dashed line indicates hydrogen-bonding interaction.

**Fig. 2.**
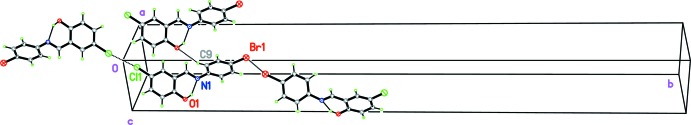
The packing diagram of the title compound, showing the intermolecular O—H···N and intermolecular Cl···Cl, Br···Br and C—H···O interactions (dotted lines).

## supplementary crystallographic information

### 1. Comment

Schiff bases are condensed by primary amines and carbonyl compounds, containing strong electronegative with atoms O and N, thus it is easy to coordinate with the metal ions to form stable complexes (Akmal *et al.*, 2007; Rehmana *et al.*, 2008). It is reported that metal complexes of Schiff base derivatives have a variety of important biological activities, such as anti-bacterial, anti-cancer, anti-tumor, hypoglycemic and so on (Vanco *et al.*, 2008; Li *et al.*, 2007; Ritter *et al.*, 2009). Our reports indicated that copper and vanadium complexes of Schiff bases are potential inhitors over protein tyrosine phosphatases (Li *et al.*, 2011; Lu *et al.*, 2011; Ma *et al.*, 2011; Yuan *et al.*, 2009, 2010).

We report here the synthesis and characterization a potentially bidentate Schiff base derivative, (I), and prepared from the condensation reaction of an equimolar proportion of 5-chloro-salicylaldehyde and 4-bromo-aniline in absolute ethanol. The molecular structure is depicted in Fig. 1. X-ray structural analysis confirmed that in the title compound, (I), the dihedral angle between the substituted benzene rings is nearly 44.25 (11)°, similar to the compound 4-bromo-2-[(*E*)-(4-chlorophenyl)iminomethyl]phenol (Ardakani *et al.*, 2011). In the crystal, there are strong intramolecular O—H···N hydrogen bonds with a distance of 2.593 (5) Å between donor and acceptor, which generate S(6) ring and intermolecular Cl1···Cl1^i^ [3.430 (2) Å, i -*x*, -*y*, -*z*] as well as Br1···Br1^ii^ [3.846 (1) Å, ii 2 - *x*, -*y*, -*z*] contacts. The crystal packing is further stabilized by intermolecular C—H···O and C—H···π interactions (Table 1).

### 2. Experimental

A 0.1566 g (1.0 mmol) 5-chloro-salicylaldehyde in 15 ml of absolute ethanol was heated until thoroughly dissolved and 0.1720 g (1.0 mmol) of 4-bromo-aniline in 5 ml of absolute ethanol was added dropwise with a constant stirring. The reaction mixture was heated under refluxing for 3 h. After cooling slowly, the orange powder was separated out. Orange–red crystal was obtained from filter after two weeks.

### 3. Refinement

H atoms attached to C and O of (I) were placed in geometrically idealized positions with C*sp*^2^—H = 0.93 Å and O—H = 0.84 Å and constrained to refine with *U*_iso_(H) = 1.2*U*_eq_(C) and *U*_iso_(H) = 1.5*U*_eq_(O).

### Crystal data

**Table:**

C_13_H_9_BrClNO	*F*(000) = 1232
*M**_r_* = 310.57	*D*_x_ = 1.720 Mg m^−^^3^
Orthorhombic, *P**c**c**n*	Mo *K*α radiation, λ = 0.71073 Å
Hall symbol: -P 2ab 2ac	Cell parameters from 4170 reflections
*a* = 6.9964 (15) Å	θ = 2.2–22.6°
*b* = 55.786 (12) Å	µ = 3.63 mm^−^^1^
*c* = 6.1443 (14) Å	*T* = 298 K
*V* = 2398.1 (9) Å^3^	Block, colourless
*Z* = 8	0.30 × 0.25 × 0.20 mm

### Data collection

**Table:**

Bruker SMART 1K CCD area-detector diffractometer	3013 independent reflections
Radiation source: fine-focus sealed tube	2056 reflections with *I* > 2σ(*I*)
Graphite monochromator	*R*_int_ = 0.061
ω scans	θ_max_ = 28.4°, θ_min_ = 2.2°
Absorption correction: multi-scan (*SADABS*; Sheldrick, 2000)	*h* = −9→9
*T*_min_ = 0.409, *T*_max_ = 0.530	*k* = −74→74
30095 measured reflections	*l* = −8→8

### Refinement

**Table:**

Refinement on *F*^2^	Primary atom site location: structure-invariant direct methods
Least-squares matrix: full	Secondary atom site location: difference Fourier map
*R*[*F*^2^ > 2σ(*F*^2^)] = 0.054	Hydrogen site location: inferred from neighbouring sites
*wR*(*F*^2^) = 0.128	H-atom parameters constrained
*S* = 1.14	*w* = 1/[σ^2^(*F*_o_^2^) + (0.027*P*)^2^ + 7.4937*P*] where *P* = (*F*_o_^2^ + 2*F*_c_^2^)/3
3013 reflections	(Δ/σ)_max_ = 0.001
155 parameters	Δρ_max_ = 0.42 e Å^−^^3^
0 restraints	Δρ_min_ = −0.95 e Å^−^^3^

### Special details

**Table:**

Geometry. All e.s.d.'s (except the e.s.d. in the dihedral angle between two l.s. planes) are estimated using the full covariance matrix. The cell e.s.d.'s are taken into account individually in the estimation of e.s.d.'s in distances, angles and torsion angles; correlations between e.s.d.'s in cell parameters are only used when they are defined by crystal symmetry. An approximate (isotropic) treatment of cell e.s.d.'s is used for estimating e.s.d.'s involving l.s. planes.
Refinement. Refinement of *F*^2^ against ALL reflections. The weighted *R*-factor *wR* and goodness of fit *S* are based on *F*^2^, conventional *R*-factors *R* are based on *F*, with *F* set to zero for negative *F*^2^. The threshold expression of *F*^2^ > σ(*F*^2^) is used only for calculating *R*-factors(gt) *etc*. and is not relevant to the choice of reflections for refinement. *R*-factors based on *F*^2^ are statistically about twice as large as those based on *F*, and *R*-factors based on ALL data will be even larger.

### Fractional atomic coordinates and isotropic or equivalent isotropic displacement parameters (Å^2^)

**Table:**

	*x*	*y*	*z*	*U*_iso_*/*U*_eq_	
Br1	−0.03618 (9)	0.228348 (8)	−0.07815 (11)	0.0634 (2)	
C1	−0.0360 (6)	0.08714 (7)	0.5859 (7)	0.0339 (8)	
C2	−0.0711 (6)	0.06455 (8)	0.6715 (7)	0.0404 (10)	
H2	−0.1186	0.0631	0.8123	0.049*	
C3	−0.0364 (6)	0.04435 (7)	0.5506 (8)	0.0426 (11)	
H3	−0.0609	0.0293	0.6092	0.051*	
C4	0.0346 (6)	0.04637 (7)	0.3425 (7)	0.0372 (10)	
C5	0.0691 (6)	0.06843 (7)	0.2537 (7)	0.0333 (9)	
H5	0.1169	0.0695	0.1129	0.040*	
C6	0.0332 (6)	0.08932 (7)	0.3722 (6)	0.0306 (8)	
C7	0.0538 (6)	0.11247 (7)	0.2675 (7)	0.0335 (9)	
H7	0.0897	0.1132	0.1219	0.040*	
C8	0.0157 (5)	0.15412 (7)	0.2599 (7)	0.0304 (8)	
C9	−0.0626 (6)	0.15603 (7)	0.0518 (7)	0.0335 (9)	
H9	−0.1044	0.1424	−0.0209	0.040*	
C10	−0.0780 (6)	0.17821 (7)	−0.0459 (7)	0.0359 (9)	
H10	−0.1315	0.1795	−0.1839	0.043*	
C11	−0.0140 (6)	0.19838 (7)	0.0610 (8)	0.0392 (10)	
C12	0.0610 (6)	0.19682 (7)	0.2681 (8)	0.0401 (10)	
H12	0.1013	0.2106	0.3402	0.048*	
C13	0.0759 (6)	0.17470 (7)	0.3676 (7)	0.0352 (9)	
H13	0.1265	0.1736	0.5072	0.042*	
Cl1	0.0736 (2)	0.02081 (2)	0.1879 (2)	0.0614 (4)	
N1	0.0235 (5)	0.13186 (6)	0.3721 (5)	0.0322 (7)	
O1	−0.0721 (5)	0.10666 (5)	0.7096 (5)	0.0474 (8)	
H1	−0.0470	0.1188	0.6401	0.071*	

### Atomic displacement parameters (Å^2^)

**Table:**

	*U*^11^	*U*^22^	*U*^33^	*U*^12^	*U*^13^	*U*^23^
Br1	0.0722 (4)	0.0352 (2)	0.0829 (4)	0.0069 (2)	−0.0019 (3)	0.0149 (3)
C1	0.035 (2)	0.038 (2)	0.029 (2)	−0.0004 (18)	−0.0007 (18)	−0.0038 (17)
C2	0.036 (2)	0.053 (3)	0.032 (2)	−0.004 (2)	0.002 (2)	0.007 (2)
C3	0.046 (2)	0.034 (2)	0.048 (3)	−0.0034 (19)	0.000 (2)	0.0120 (19)
C4	0.039 (2)	0.0318 (19)	0.041 (2)	0.0016 (18)	−0.001 (2)	−0.0023 (17)
C5	0.037 (2)	0.0304 (19)	0.032 (2)	0.0017 (17)	0.0008 (19)	−0.0019 (16)
C6	0.030 (2)	0.0345 (19)	0.028 (2)	0.0024 (17)	−0.0034 (17)	0.0023 (15)
C7	0.034 (2)	0.038 (2)	0.029 (2)	0.0013 (17)	0.0027 (19)	0.0018 (17)
C8	0.029 (2)	0.0299 (18)	0.032 (2)	0.0004 (15)	0.0030 (17)	−0.0019 (16)
C9	0.039 (2)	0.0289 (18)	0.033 (2)	−0.0029 (17)	−0.0012 (19)	−0.0041 (16)
C10	0.033 (2)	0.038 (2)	0.037 (2)	0.0031 (17)	0.0000 (19)	−0.0019 (18)
C11	0.036 (2)	0.0315 (19)	0.050 (3)	0.0041 (17)	0.006 (2)	0.0035 (19)
C12	0.042 (2)	0.0299 (19)	0.048 (3)	−0.0017 (18)	−0.003 (2)	−0.0077 (18)
C13	0.033 (2)	0.037 (2)	0.036 (2)	−0.0005 (17)	−0.0029 (18)	−0.0080 (17)
Cl1	0.0858 (10)	0.0333 (5)	0.0652 (8)	−0.0007 (6)	0.0074 (8)	−0.0081 (6)
N1	0.0343 (18)	0.0305 (15)	0.0318 (18)	−0.0003 (14)	−0.0008 (15)	−0.0009 (14)
O1	0.069 (2)	0.0394 (16)	0.0342 (17)	0.0003 (16)	0.0096 (17)	−0.0042 (13)

### Geometric parameters (Å, º)

**Table:**

Br1—C11	1.884 (4)	C7—H7	0.9300
C1—O1	1.352 (5)	C8—C13	1.390 (5)
C1—C2	1.387 (6)	C8—C9	1.395 (6)
C1—C6	1.405 (6)	C8—N1	1.422 (5)
C2—C3	1.371 (6)	C9—C10	1.379 (5)
C2—H2	0.9300	C9—H9	0.9300
C3—C4	1.377 (6)	C10—C11	1.378 (6)
C3—H3	0.9300	C10—H10	0.9300
C4—C5	1.367 (6)	C11—C12	1.379 (7)
C4—Cl1	1.735 (4)	C12—C13	1.381 (6)
C5—C6	1.397 (5)	C12—H12	0.9300
C5—H5	0.9300	C13—H13	0.9300
C6—C7	1.450 (5)	O1—H1	0.8200
C7—N1	1.276 (5)		
			
O1—C1—C2	119.1 (4)	C13—C8—C9	119.5 (4)
O1—C1—C6	121.3 (4)	C13—C8—N1	118.6 (4)
C2—C1—C6	119.6 (4)	C9—C8—N1	121.7 (3)
C3—C2—C1	120.6 (4)	C10—C9—C8	119.8 (4)
C3—C2—H2	119.7	C10—C9—H9	120.1
C1—C2—H2	119.7	C8—C9—H9	120.1
C2—C3—C4	120.0 (4)	C11—C10—C9	120.0 (4)
C2—C3—H3	120.0	C11—C10—H10	120.0
C4—C3—H3	120.0	C9—C10—H10	120.0
C5—C4—C3	120.5 (4)	C10—C11—C12	120.8 (4)
C5—C4—Cl1	119.6 (3)	C10—C11—Br1	118.8 (3)
C3—C4—Cl1	119.9 (3)	C12—C11—Br1	120.4 (3)
C4—C5—C6	120.8 (4)	C11—C12—C13	119.6 (4)
C4—C5—H5	119.6	C11—C12—H12	120.2
C6—C5—H5	119.6	C13—C12—H12	120.2
C5—C6—C1	118.5 (4)	C12—C13—C8	120.3 (4)
C5—C6—C7	119.6 (4)	C12—C13—H13	119.9
C1—C6—C7	121.7 (4)	C8—C13—H13	119.9
N1—C7—C6	121.0 (4)	C7—N1—C8	120.2 (3)
N1—C7—H7	119.5	C1—O1—H1	109.5
C6—C7—H7	119.5		
			
O1—C1—C2—C3	179.9 (4)	C1—C6—C7—N1	5.4 (6)
C6—C1—C2—C3	0.9 (6)	C13—C8—C9—C10	0.6 (6)
C1—C2—C3—C4	0.3 (7)	N1—C8—C9—C10	176.0 (4)
C2—C3—C4—C5	−0.8 (7)	C8—C9—C10—C11	0.7 (6)
C2—C3—C4—Cl1	−178.6 (4)	C9—C10—C11—C12	−1.8 (6)
C3—C4—C5—C6	0.1 (6)	C9—C10—C11—Br1	179.7 (3)
Cl1—C4—C5—C6	178.0 (3)	C10—C11—C12—C13	1.4 (7)
C4—C5—C6—C1	1.1 (6)	Br1—C11—C12—C13	179.9 (3)
C4—C5—C6—C7	−174.2 (4)	C11—C12—C13—C8	0.0 (6)
O1—C1—C6—C5	179.5 (4)	C9—C8—C13—C12	−1.0 (6)
C2—C1—C6—C5	−1.6 (6)	N1—C8—C13—C12	−176.6 (4)
O1—C1—C6—C7	−5.3 (6)	C6—C7—N1—C8	−170.4 (3)
C2—C1—C6—C7	173.6 (4)	C13—C8—N1—C7	−148.7 (4)
C5—C6—C7—N1	−179.4 (4)	C9—C8—N1—C7	35.8 (6)

### Hydrogen-bond geometry (Å, º)

Cg1 and Cg2 are the centroids of the C1–C6 and C8–C13 benzene rings, respectively.

**Table:**

*D*—H···*A*	*D*—H	H···*A*	*D*···*A*	*D*—H···*A*
O1—H1···N1	0.82	1.87	2.593 (4)	147
C2—H2···*Cg*1^i^	0.93	2.82	3.489 (5)	129
C5—H5···*Cg*1^ii^	0.93	2.85	3.513 (5)	129
C10—H10···*Cg*2^iii^	0.93	2.75	3.460 (5)	133
C13—H13···*Cg*2^iv^	0.93	2.78	3.473 (5)	132

Symmetry codes: (i) −*x*+1/2, *y*, *z*−1/2; (ii) −*x*+3/2, *y*, *z*−3/2; (iii) −*x*+1/2, *y*, *z*−3/2; (iv) −*x*+3/2, *y*, *z*−1/2.
